# Cost-utility analysis of hearing aid device for older adults in the community: a delayed start study

**DOI:** 10.1186/s12913-020-05977-x

**Published:** 2020-12-01

**Authors:** Palvinder Kaur, Sheue Lih Chong, Palvannan Kannapiran, W.-S. Kelvin Teo, Charis Ng Wei Ling, Chiang Win Weichen, Gan Ruling, Lee Sing Yin, Tang Ying Leng, Soo Ying Pei, Then Tze Kang, Lim Zhen Han, Lin Peizhen, Lynne Lim Hsueh Yee, Pradeep Paul George

**Affiliations:** 1 Health Services and Outcomes Research, National Healthcare Group, 3 Fusionopolis Link #03-08 Nexus@One-north, Singapore, 138543 Singapore; 2 Ear, Nose and Throat Head & Neck Department, National University Hospital, 5 Lower Kent Ridge Road, Singapore, 119074 Singapore; 3 Ear Nose Throat & Hearing Centre, 3 Mount Elizabeth, Singapore, 228510 Singapore

**Keywords:** Cost-utility analysis, Hearing aids, Singapore, Community, Cost-effectiveness, Older adults, Delayed-start

## Abstract

**Background:**

Hearing aids (HA) is the primary medical intervention aimed to reduce hearing handicap. This study assessed the cost-effectiveness of HA for older adults who were volunteered to be screened for hearing loss in a community-based mobile hearing clinic (MHC).

**Methods:**

Participants with (1) at least moderate hearing loss (≥40 dB HL) in at least one ear, (2) no prior usage of HA, (3) no ear related medical complications, and (4) had a Mini-Mental State Examination score ≥ 18 were eligible for this study. Using a delayed-start study design, participants were randomized into the immediate-start (Fitted) group where HA was fitted immediately or the delayed-start (Not Fitted) group where HA fitting was delayed for three months. Cost utility analysis was used to compare the cost-effectiveness of being fitted with HA combined with short-term, aural rehabilitation with the routine care group who were not fitted with HA. Incremental cost effectiveness ration (ICER) was computed. Health Utility Index (HUI-3) was used to measure utility gain, a component required to derive the quality adjusted life years (QALY). Total costs included direct healthcare costs, direct non-healthcare costs and indirect costs (productivity loss of participant and caregiver). Demographic data was collected during the index visit to MHC. Cost and utility data were collected three months after index visit and projected to five years.

**Results:**

There were 264 participants in the Fitted group and 163 participants in the Not Fitted group. No between-group differences in age, gender, ethnicity, housing type and degree of hearing loss were observed at baseline. At 3 months, HA fitting led to a mean utility increase of 0.12 and an ICER gain of S$42,790/QALY (95% CI: S$32, 793/QALY to S$62,221/QALY). At five years, the ICER was estimated to be at S$11,964/QALY (95% CI: S$8996/QALY to S$17,080/QALY) assuming 70% of the participants continued using the HA. As fewer individuals continued using their fitted HA, the ICER increased.

**Conclusions:**

HA fitting can be cost-effective and could improve the quality of life of hearing-impaired older individuals within a brief period of device fitting. Long term cost-effectiveness of HA fitting is dependent on its continued usage.

**Supplementary Information:**

The online version contains supplementary material available at 10.1186/s12913-020-05977-x.

## Background

As we age, our sensory abilities may decline [1]. Presbycusis or age related hearing loss is a prevalent health problem in ageing societies [2]. The latest global burden of disease (GBD) study reported age related hearing loss to be the third leading cause of years lived with disability (YLD) [3]. Clinically meaningful hearing loss and disabling hearing loss in adults is regarded as hearing loss greater than 25 and 40 dB hearing level (dB HL) averaged over four frequencies of 0.5, 1, 2 and 4 Hz in the better ear, respectively [4]. Globally, an estimated 434 million adults experience disabling hearing loss, with nearly one third over the age of 65 years [4]. More than 38 million adults in United States (US) experience hearing loss (> 25 dB HL), of which two-thirds of the adults were over the age of 70 years [5–7]. Due to ageing population trends, the global prevalence of older adults with hearing loss is projected to double by year 2060 [4, 6, 7].

Singapore, a small country with a population size of about 5.6 million, has one of the fastest ageing population in the world. By 2035, it is projected that 32% of the population will be aged 65 years and above [8]. In 2010, the reported prevalence of at least mild hearing loss (≥25 dB HL) and at least moderate hearing loss (≥40 dB HL) in the better ear, among residents aged between 18 and 69 years old was 11.6 and 2.9% respectively [9]. With increasing age, the prevalence of hearing loss increased linearly. For the elderly aged between 60 to 69, the prevalence of at least mild hearing loss and moderate hearing loss was 32.9 and 9.6% respectively [9]. In 2017, a community-based study estimated the national prevalence of hearing loss and disabling hearing loss among Singapore residents over 60 years of age to be 63.7 and 16.2% respectively [10]. Mirroring global trends, the prevalence of age-related hearing impairment in Singapore is expected to increase in the future.

Hearing loss has profound negative impact on the individual, significant others, and society. It is associated with multiple negative health outcomes such as functional and cognitive impairment [11, 12], comorbidities [13], poor quality of life [14] and increased healthcare utilization and costs [15, 16]. The impact of reduced hearing ability may also have a collateral effect on interpersonal relationship, as communication with others become challenging [17]. Adults with hearing loss were reported to have lower employment rates and reduced work productivity as compared to adults with normal hearing [18, 19]. A recent systematic review in US found that the direct medical costs due to hearing loss ranged between US$3.3 billion to US$12.8 billion, while the economic cost of productivity loss varied widely between US$1.8 billion to US$194 billion [20]. In Singapore, the estimated cost of hearing loss of any degree (> 25 dB HL) in 2013 was approximately Singapore dollar (SGD) $1.1 billion and predicted to increase by 53% by year 2030 [21]. Productivity loss due to hearing impairment was projected to be approximately SGD$986 million, which accounted for 0.33% of the growth domestic product (GDP) in 2013 [21].

The most common clinical intervention for people with age-related hearing loss is hearing aids (HA). These medical devices do not restore hearing; however, they make sounds more audible through electroacoustic amplifications. The goal of HA is to reduce hearing handicap and promote a more active and engaged lifestyle which as a result, improves general quality of life, interpersonal relationships, emotional and physical functioning in adults with mild to moderate hearing loss [22–24]. Following HA fitting, aural rehabilitation services may be offered to help relearn hearing with the device. Despite the known benefits, a large majority of those who require HA do not use them. This reluctance is contributed mainly by device related factors (maintenance, fit, comfort and cost) and non-device related factors (perception and severity of deafness, lack of awareness of hearing loss, stigma, healthcare professionals and HA user attitudes) [25]. Evidence suggest that hearing loss in older adults tend to be underdiagnosed and undertreated [26, 27]. Up to 73.2% of residents with at least moderate hearing loss indicated that they were not aware that they had hearing loss and only 3.3% reported wearing HA [9]. In Singapore, HA are typically obtained from Ear, Nose and Throat (ENT) clinics or by audiologists in the private sector. In the public sector, individuals with hearing loss will require a referral from the polyclinics to ENT specialist outpatient clinic (SOC). Multiple visits are required before HA can be fitted, with follow-up sessions with an audiologist post HA fitting. To improve access to and awareness on hearing healthcare, two units of mobile hearing clinics (MHC) were deployed to the community in December 2015. Residents could obtain hearing tests, HA fitting and aural rehabilitation in these community-based MHCs.

Cost utility analysis (CUA) is the recommended method used to perform economic evaluations. It compares the health benefit, typically expressed as the patient-reported utility, quality adjusted life years (QALY), to the cost of an intervention. This cost utility ratio assesses interventions by what health related improvements can be achieved per unit cost. Information regarding cost effectiveness of HA is scarce. A recent review of seven studies that performed economic evaluation of HA (digital or analogue) found that there was high variability in the cost-effectiveness of the HA device ranging from USD 9702 to USD 721,942/QALY [28]. Only three studies accounted for productivity loss due to hearing impairment [29–31]. There is no information on the cost-effectiveness of HA device in Singapore. The objective of this study was to determine the cost effectiveness of fitting hearing aids and aural rehabilitation in an adult population with hearing loss compared with the alternative of not being fitted with a hearing aid, in the MHC setting.

## Methods

### Population

Participants were residents who volunteered to be screened for hearing loss at a community-based mobile hearing clinic (MHC) from December 2015 to June 2017. They are likely to be those who suspect that they may have some hearing issue or want to assure themselves that they are fine. Participants who were found to have (1) at least moderate hearing loss (≥40 dB HL) in at least one ear, (2) no prior usage of HA in the last ten years and (3) free from ear related medical complications and (4) had a Mini-Mental State Examination (MMSE) score ≥ 18 were eligible for this study. Eligible participants were then offered the HA services in the MHC.

### MHC

The MHC is a community outreach program staffed by a team of audiologists, research assistants and trained audio technicians that offers hearing diagnostic tests, referrals general practitioners (GP) or ENT specialist clinic, HA fitting and short-term post-fitting audiological rehabilitation services. Sited in a purpose-built truck chassis, the MHC has been specially retrofitted with two sound-treated rooms and audiological equipment for the delivery of hearing tests, HA fitting and aural rehabilitation. During the study, the MHC was parked in 38 community centres or near-by neighbourhoods to provide hearing test and HA fitting services to residents.

### Hearing screening at MHC

Research assistants and audiologists screened participants for hearing loss through a series of steps. Otoscopy examination was performed by research assistants to inspect for outer ear conditions such as impacted ear cerumen, ear infection, perforated tympanic membrane and presence of any foreign body in the ear canal [32]. A trained audio technician or audiologist examined middle ear function using tympanometry to detect ear related medical complications such as ear infection, otitis media and middle ear effusion [33]. Pure tone audiometry (PTA) test was then performed to determine hearing levels at 0.25 kHz to 8 kHz. To facilitate the categorization of hearing loss severity, the pure tone average was computed by averaging hearing levels at 0.5, 1, 2 and 4 kHz [34]. Participants with moderate hearing loss in at least one ear, i.e. PTA ≥40 dB HL were recruited into the study. Participants were also screened for cognitive impairment using the MMSE questionnaire.

Participants with impacted ear cerumen or ear related medical complication were referred to general practitioners or public hospitals and were excluded from the study. Participants with MMSE scores < 18 or those with prior usage of HA in the last 10 years were also excluded from the study.

### HA selection, Fitting & Aural Rehabilitation at MHC

After hearing screening, eligible participants who were keen to adopt HA were scheduled for HA selection based on treatment groups they were randomly assigned to (please see study design). During the HA selection session, audiologist discussed with study participant his/her hearing test result, importance, benefits, limitations, and the different types of HA. HA use in both ears were encouraged for participants with binaural hearing loss. Upon decision to adopt HA use and participate in the study, enrolled study participants were then scheduled for HA fitting within the following two weeks.

HA fitting was conducted by an audiologist in accordance to the American Speech Language Hearing Association (ASHA) HA fitting guidelines [35]. Physical fit of HA was checked by the audiologist and verified using a probe microphone measurement. To ensure competency in HA use, participants were briefed on usage, care, and maintenance of the device. Participants practiced HA insertion and removal, turning device on and off, adjusting volume, battery changing and cleaning of HA. Simplified user manual and leaflet that detailed device use, care and maintenance was also provided.

Aural rehabilitation was scheduled two weeks post HA fitting, in a small group setting at the community centre. The aim of the rehabilitation was to re-emphasize use, care, and maintenance of HA, re-learn hearing with the use of the device, practise communication skills and repair strategies and clarify/discuss any issues participants were having with regards to HA use. Two additional rehabilitation sessions were given to participants at one- and three-months post HA fitting. Participants that required more sessions could make an appointment at the MHC.

### Study design

To establish effectiveness of the HA intervention, suitable controls were required. Residents with hearing loss and refused HA may not be viable controls as there may be systematic differences (e.g. socioeconomic differences) between those who took up the HA and those who did not. An ideal control group would be eligible participants for whom we can delay the HA intervention and rehabilitation.

**Fig. 1:**
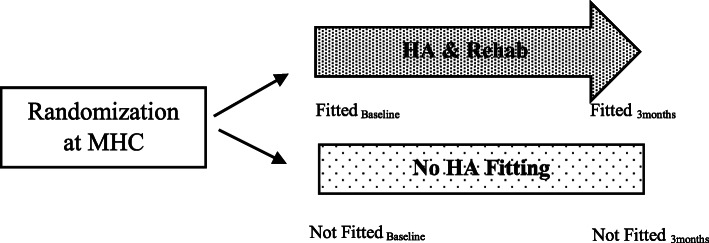
Study design for the economic evaluation of HA at community-based mobile health clinic (MHC) in Singapore

A prospective delayed-start randomized design was used for this study [36]. Participants who had met the inclusion criteria of this study were randomized into one of the following two groups – (1) immediate-start (Fitted) group where HA was fitted two weeks after HA selection or (2) delayed-start (Not Fitted) group where HA selection and fitting was delayed for three months (Fig. 1). After three months, the Not Fitted group were scheduled for HA selection, fitting and followed-up with aural rehabilitation services at the MHC like the Fitted group.

Participant’s demographic, employment status and occupation information were collected during the participant’s first visit to the MHC. Outcomes were collected for both Fitted and Not Fitted groups at baseline and three months (Fig. 1). For this economic evaluation, the study outcomes of the Fitted group were compared with the Not Fitted group three months after the index visit to the MHC (Fig. 1).

### Randomization

Using the RANDBETWEEN(0,1) function in Microsoft Excel Version 2004, community centres with random number “0” were assigned to the immediate treatment group, while the community centres with random number “1” were allocated to the delayed treatment group. The MHC was assigned to each community centre on a weekly basis.

### Sample size calculation

To detect a difference in health-related quality of life (HRQoL) as low as 0.06 [37] with an expected standard deviation of 0.2, statistical power of 80%, significance level of 5% and an attrition rate of 20%, a total sample size of 480 participants was required for Fitted (*n* = 240) and Not Fitted (n = 240) groups.

### Cost utility analysis

Cost utility analysis (CUA) was used to compare the cost-effectiveness of being fitted with a HA combined with short-term post audiological rehabilitation (Fitted) with the control group who received no treatment (Not Fitted). Two outcomes were measured for this study – (1) patient-reported utility, using the quality adjusted life years (QALYs) metric and (2) total costs. QALY is a measure of health as a combination of duration of life and health related quality of life (HRQoL). Health utility index (HUI-3) is a psychometric questionnaire that this study’s participants completed in order to measure change in HRQoL. The HUI-3 comprises of eight attributes – vision, hearing, speech, ambulation, dexterity, emotion, cognition, and pain. Each attribute has about 5 to 6 levels of ability or disability. A combination of levels across the eight attributes constitutes as a health state. The health state is converted into a utility score of 0 (dead) to 1 (perfect health). Due to the inclusion criteria of no HA usage prior to study enrolment and upon advice from the tool developers, the HUI-3 was modified to exclude questions regarding HA use (see additional file 1 Table S1 for modified scoring methodology). The modified HUI-3 was administered to the Not Fitted group at three months. HUI-3 scores were expressed as mean ± SD.

### Estimation of costs

**Fig. 2:**
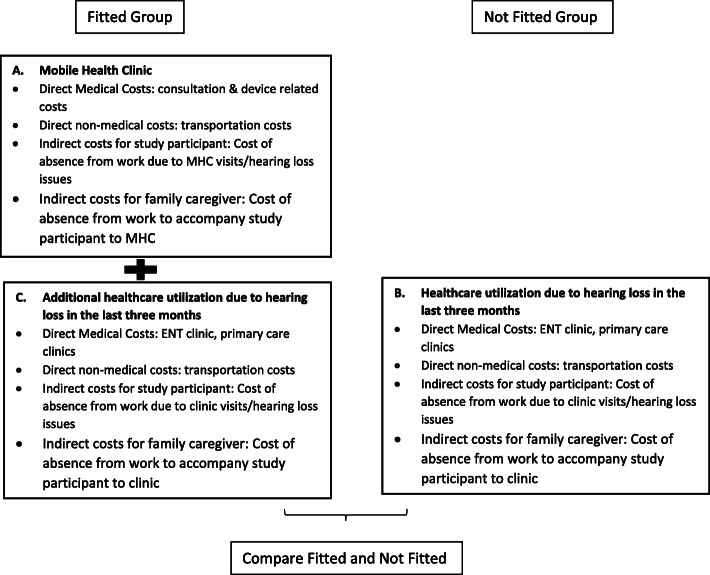
Estimation of costs for Fitted and Not Fitted groups

Costs were estimated from a societal perspective and included direct healthcare, direct non-healthcare, and indirect costs **(**Fig. 2**)**. Direct healthcare costs were defined as the expenditures incurred in the healthcare management system for the diagnosis, treatment, and management of hearing loss. In this study, the direct healthcare costs included:

1. All expenditures incurred at the MHC due to hearing loss and
2. All healthcare expenditures incurred due to visits to any other healthcare institutions (such as ENT specialist clinics or primary care clinics) due to hearing loss, three months after the index visit to the MHC.

Expenditure incurred at the MHC included consultation-related (hearing loss evaluation, HA fitting, rehabilitation, and hearing tests) and device-related (HA device, maintenance and repair and consumables) costs. Using a cost questionnaire developed by the study team, (please refer to additional file 2), participants were also asked through face to face interviews to recall the number of visits made to any healthcare institutions due to hearing loss in the last three months. The number of units consumed was then multiplied by the norm cost of each visit (additional file 1 Table S2). To capture direct non-healthcare related costs, participants were asked to estimate transportation costs incurred during visits to MHC and/or visits to any other healthcare institutions in the last three months due to hearing loss.

Indirect costs were defined as productivity loss due to hearing impairment Besides study participants, we accounted for productivity losses incurred by working family caregivers who may have taken time off from work to accompany study participants to the MHC and/or other healthcare institutions. Family caregiver was defined as informal care that was provided by family members. Productivity losses for participants and family caregiver were measured in units in time (days) and monetised using the human capital approach [38]. The method of estimating indirect costs were different for working and non-working adult participants. Working participants were asked to estimate the number of days absent from work due to hearing impairment in the three months after the first visit to MHC. Indirect costs were calculated by multiplying number of days absent from work due to hearing loss multiplied by the median earnings (based on individual’s occupation) per capita per day. For retirees or home makers, the estimated number of days taken to visit MHC and/or any healthcare institution due to hearing loss was multiplied by the median market wage for housekeeping (i.e. SGD 550). During the face-to-face interviews at the three-month timepoint, study participants were asked if a working family caregiver accompanied them to the MHC and/or other healthcare institutions during the last three months. Information on occupation of the working family caregiver was recorded, and similar methods was used to calculate productivity losses.

### Statistical analysis

Descriptive analysis was used to compare the baseline characteristics of Fitted and Not Fitted Groups. Costs for Fitted group (MHC costs, healthcare costs, transport costs and productivity losses) were compared with Not Fitted group (healthcare costs, transport costs and productivity losses) **(**Fig. 2**)**. Costs were expressed as mean costs (standard deviation) per patient in 2017 Singapore dollar.

The incremental cost effectiveness ratio (ICER), otherwise known as cost per QALY, was the primary outcome measure of this CUA. ICER was computed by taking the difference between costs of the groups divided by the difference in QALY produced by the two groups. Health interventions with a cost per QALY of < 50,000 USD/QALY is cost effective [39]. As cost per QALY decreases, the intervention becomes more cost effective.

ICER was computed based on the primary data and then extrapolated to five years with the following assumptions:

1. 25, 50 and 70% of the participants continued using the HA for five years
2. Utility gained at three months was constant for five years
3. Healthcare utilization and productivity losses incurred at three months for both groups was projected for five years with a 5% discount rate.

As part of the services of the MHC, participants were followed up for one year. Through telephone interviews, research assistants asked the Fitted group participants if they were still using the HA **(**additional file 1 Table S3). With a response rate of 66, 71.4% were still using the device after one year. Currently, there is no evidence of prolonged utility gain over time after HA intervention. Based on the National Institute for Health and Care Excellence (NICE) guidelines, a constant rate of benefit to quality of life for everyone using HA, regardless of age, duration of HA use or level of hearing loss can be assumed [40]. A local study identified that the average usage period of the device to be five years [41]. Hence, we assumed constant utility over the five-year usage period of the device.

To establish a 95% Confidence Interval (CI) for point estimates of ICER, bootstrapping was performed that generated 1000 predicted values for the cost-utility ratio. Similar scenarios were projected with 50 and 25% of participants using HA for 5 years. The computation of costs and QALY were done using Microsoft Excel Version 2004. All other analysis was conducted using R statistical software version 3.4.

## Results

A summary of population profile can be found in Table 1. A total of 3657 residents were screened at the MHC between December 2015 and June 2017, of which, 439 residents met the study criteria and were keen on adopting HA. Twelve participants (10 from Fitted, and two from Not Fitted group had missing HUI-3 or cost data and were excluded from the analysis. There were 264 participants who were enrolled into the Fitted group and 163 participants in the Not Fitted group. Mean age at enrollment was 69 (SD: 10.8) years for both groups. There were no statistical differences in the distribution of ethnicity, gender housing type and degree of hearing loss between the two groups **(**Table 1**)**. Approximately 62% of participants were retired, 34% were employed and 4% were unemployed in both groups. The employment status of the caregiver are as follows: 49% employed; 30% were retired; 14.1% were unemployed, 3.9% were foreign domestic workers (FDWs) and 3% others, with no differences in the distribution between the two groups.

**Table 1:** Baseline Characteristics of Participants Fitted with Hearing Aid and Participants Not Fitted with Hearing Aid

Characteristics	Fitted (***n*** = 264)	Not Fitted (163)	***P*** value
**Age (years) (mean, SD)**	69.3 (10.8)	69.5 (10.9)	0.86
**Ethnicity (n, col %)**			
Chinese	248 (93.9)	155 (95.7)	
Malay	4 (1.5)	4 (2.5)	
Indian	9 (3.4)	2 (1.2)	
Others	3 (1.1)	1 (0.6)	0.45
**Gender**			
Male	161 (61.0)	94 (58.0)	
Female	103 (39.0)	68 (42.9)	0.54
**Housing type**			
Public	206 (78.0)	135 (83.3)	
Private	58 (22.0)	27 (16.7)	0.38
**Degree of hearing loss (dB HL) (mean, SD)**			
Poorer ear	59.8 (13.2)	60.6 (13.4)	0.54
Better ear	52.0 (12.3)	53.7 (10.5)	0.16
**Employment status of participants**			
Retired	165 (62.5)	101 (62.0)	
Employed (full-time/part-time)	89 (33.7)	55 (33.7)	
Unemployed	10 (3.8)	7 (4.3)	0.96
**Number of employed participants who were absent from work when visiting MHC**	35 (39.3)	19 (34.5)	0.56
**Number of participants who had caregiver during visit to MHC**	74 (28.0)	53 (32.5)	0.30
**Employment status of caregivers**			
Employed	37 (50.0)	26 (49.1)	
Retired	21 (28.4)	17 (32.1)	0.80
Unemployed	10 (13.5)	8 (15.1)	
Domestic helper	3 (4.1)	2 (3.8)	
Others	3 (4.1)	0	
**Number of participants who had visited healthcare institution due to hearing loss in the last three months**	37 (14.0)	25 (15.3)	0.71
**Mean number of visits for participants who had visited any healthcare institutions in the last three months**	1.0 (0.16)	1.20 (0.5)	0.05
**Number of employed participants who were absent from work when visiting healthcare institution**	7 (18.9)	7 (28.0)	0.40
**Mean number of days absent from work for participants who had visited any healthcare institutions**	0.32 (0.9)	0.44 (0.8)	0.60
**Number of participants with caregivers when visiting any healthcare institutions**	7 (18.9)	9 (36.0)	0.13
**No. of employed caregivers who were absent from work when visiting healthcare institution**	0.6 (0.53)	0.6 (0.5)	0.95

At the MHC, the direct costs incurred for the Fitted group was SGD $4809.50 which includes consultation and device costs **(**Table 2**)**. Direct non-healthcare cost which was mainly transportation costs amounted to an average of $37.95. For the Fitted group, 39.3% of participants were employed and were absent from work for at least an average of 4.4 (SD:2.0) days due to visits to the MHC. Less than one-third of these participants were accompanied by a caregiver during the visit to MHC (*n* = 74). Only 37 caregivers were employed, of which nearly 50% had taken time from work to accompany the participant to the MHC and were absent from work for an average of 4.9 (SD:2.1) days. This resulted in a total productivity loss of participant and caregiver of $168.90.

**Table 2:** Breakdown of the direct medical cost incurred at the MHC for 5 years

Cost Type	Description	Estimated Cost (SGD)	Frequency	Subtotal (SGD)
Consultation	HA evaluation	99.50	Once in 5 years	99.50
	HA fitting	75.00	Once in 5 years	75.00
	HA follow-up (non-subsidised)	50.00	Twice for first year; once for subsequent years	300.00
	Rehabilitation^a^ (optional)	62.00	5 sessions	0
	Hearing test	33.00	Once in 5 years	165.00
	Consultation (non-subsidised)	100	Once in 5 years	100.00
Device cost	HA device	3000.00	Once in 5 years	3000.00
	HA maintenance and repair	200.00	Twice in 5 years	400.00
	HA consumables (batteries/drying capsules etc.)	134.00	Once per year	670.00
Total				4809.50

^a^ Rehabilitation costs were not included in the final cost as it is considered as optional

For additional healthcare utilization due to hearing loss that occurred during the three months after index visit to the MHC, the total costs were minimal. Less than 20% of the participants in both groups had at least one visit to any healthcare institutions due to hearing loss in the three months after index visit to MHC. Of which, seven patients were absent from work for approximately 0.5 days for each group. Only seven caregivers in Fitted and nine caregivers in Not Fitted group were present during these visits with only less than half day of leave. The total costs for both groups amounted to less than $35 (Fitted group: $25.30; Not Fitted group: $32.60).

**Fig. 3:**
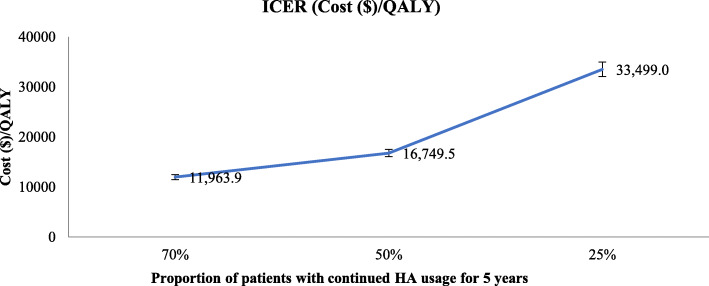
ICER with varying proportions of participants that continued use of HA over 5 years

HUI-3 scores computed for all timepoints is provided in additional file 1 Fig. S1. Table 3 summarises the total cost and mean utility scores for both groups. HA device produced a clinically significant mean utility increase of 0.12 over 3 months. ICER for 3 months was $42,790/QALY (Table 3). ICER was $11,964/QALY for 5 years with a 30% drop out rate. As fewer participants continued usage of HA for 5 years, the ICER increased (Fig. 3).

**Table 3:** Total costs and mean utility scores produced from Fitted and Not Fitted Groups

	Total Cost ($)		Utility Score		ICER ($/QALY) (95% Confidence Interval)
	Fitted	Not Fitted	Fitted	Not Fitted	
**Mean Estimates**	5041.70	32.60	0.77	0.65	42,790 (32,794 to 62,222)

## Discussion

This prospective study indicates that moderately hearing-impaired individuals achieved significantly improved HRQoL within a brief period following HA fitting. The ICER determined by this study was within the threshold of cost-effectiveness for older adults being fitted with HA coupled with short-term audiological rehabilitation at the community-based MHC in Singapore. However, as the proportion of participants who continued with the use of HA over the projected 5 years decreased, the ICER increased. Most of the participants in both Fitted and Not Fitted group were either retired or unemployed. Considering the age distribution of this population, this finding seems reasonable. Healthcare utilization and productivity loss (participant and caregiver) due to hearing loss were low for both groups. Therefore, being fitted with HA would have minimal impact on productivity gain in this population.

The limited studies that assessed the cost-effectiveness of HA compared to no HA resulted in a wide range of ICERs (USD9702 to USD721,942/ QALY) [28]. These studies employed various study design such as dynamic patient flow model, pre-post design, Markov state-transition model, WHO-CHOICE method and observational design to ascertain cost-effectiveness [28]. The high variability in the ICERS can be explained by utility measurements. The magnitude of utility gain obtained from these diverse utility measurements has significant impact on the cost effectiveness of HA. Grutters et al compared the utility scores, utility gain and ICERS derived from EQ5D (UK and Dutch tariff) and HUI (HUI-2 and HUI-3) in a cohort of patients with hearing complaints before and after being fitted with a HA [42]. After being fitted with HA, a significant improvement in utility was seen in HUI-2 (+ 0.07) and HUI-3 (+ 0.12), unlike EQ5D (UK: + 0.01; Dutch:0.00). The corresponding impact on ICERS were as follows: €25,337/QALY (HUI-2), €15,811/QALY (HUI-3), €286,866/QALY (EQ5D UK tariff) and €647,209/QALY (EQ5D Dutch tariff) [42]. In a similar study that compared utility scores pre and post HA fitting, the mean utility improvement in HUI-3 was + 0.06 points. This increase in utility in such a short time period could be attributed to the responsiveness of the tools used to measure utility. EQ5D and HUI-3 differs in the domains of health that they measure and scoring methodology. EQ5D focuses on current general health status and its impact on physical, mental, and social functioning. HUI measures underlying level of impairment and includes sensory dimensions (vision, speech, and hearing). EQ5D may not be able to capture any changes pre and post HA fitting due to the lack of sensory domains, thus rendering it unresponsive to measure HRQoL change in people with hearing loss. Due to these differences, the utility gains captured by these instruments can be highly variable and can lead to a wide range of cost-effectiveness results when applied to economic evaluations. Although the National Institute for Health and Care Excellence (NICE) recommends the use EQ5D for economic evaluations, HUI-3 has been shown to be more responsive and appropriate to use in a population with hearing loss. Therefore, the HUI-3 was the instrument of choice for this population.

Being fitted with HA not only reduces hearing handicap but also improves the emotional, social and physical function of adults with hearing loss [22–24]. Despite the increasing prevalence of hearing loss, the uptake of HA is low. Less than 20% of adults with hearing loss reported uptake of HA in the U.S. [7]. In Singapore, between 3.3 to 7.5% of adults with disabling hearing impairment reported uptake of HA [9, 10]. Even if adults were fitted with HA, not all use the HA or wear them regularly or are satisfied with the device. Non-use of HA is a waste of resources (time, money, and manpower) for both the individual and the healthcare system. Several barriers to understand reasons for the low uptake of HA has been identified [25, 43]. Prevalence and severity of age-related hearing loss increases progressively from the age of 55 [44]. Despite this onset age, HA are only adopted for the first time 15 to 20 years later when the hearing impaired individual reaches mid-70s [22]. Hearing loss is often trivialised as part of the ageing process. People get accustomed to the reduced hearing ability until they cannot hear and must seek medical help. The older the first-time HA user gets, the greater the difficulty in adopting and maintaining them. Other barriers to HA use include stigmatization, severity of hearing loss, personality factors such as attitude towards HA and motivation, low trust in HA benefit, cost, HA fit and comfort, appearance, and maintenance [43]. Similar reasons were cited in a Singaporean study conducted on geriatric patients with hearing loss [45]. Interventions such as follow-up counselling services to HA-users found increase in the uptake and regular use of HA which improved the cost effectiveness of the device [46]. Fitting HA at an earlier age not only improves health outcomes for the individual but also makes the device more cost effective as this contributes to more years of effective use of HA [47]. Effective strategies are urgently required to promote the management of patients with hearing loss at an earlier age.

This is the first study to have evaluated the economic costs of HA in the context of a community-based MHC in Singapore. Individuals who refused to take up the HA may not be suitable as controls as there could be systematic differences such as socioeconomic status between those who took up and did not take up HA. The delayed start study design facilitated selection of suitable controls that were similar in characteristics to participants who were willing to take up the HA by delaying treatment of HA fitting and rehabilitation for three months so study outcomes can be collected. However, the sample size for the Not Fitted group was lower than what was computed resulting in a study power of 77%. Despite the smaller sample size, there were no differences in the profile of participants in Fitted and Not Fitted group.

One of the main limitations of this study was that this study was based on volunteers who had come to the MHC for screening and may not represent the general populations. Subjects that came for screening were mostly retied which may have allowed them to visit the MHC. Productivity loss computed from study population may also not be generalizable to adults who are still in the working force. Participants were also asked to recall the number of visits to the clinic due to hearing loss, transportation costs and number of days absent from work (for both participant and caregiver) in the last three months after the first visit to the MHC. This could have resulted in underestimated costs and healthcare utilization due to recall bias. Short-term economic costs of fitting older adults with moderate to severe hearing loss with HA at the MHC as well as healthcare utilization at other primary care or specialist clinics were considered for this study. It assumes all participants fitted with HA were satisfied users and did not account for all other possible transitions that may occur for individuals with hearing loss such as satisfied HA-users and dissatisfied HA-users, progression of hearing loss or even frequency of HA use. Satisfied HA-users may use HA regularly and be compliant with routine check-ups while the dissatisfied HA-users may revisit audiologists to resolve issues regarding use of HA, which may result in different healthcare costs [28]. There are known health benefits that comes with HA use [48]. Research shows that use of HAs among older adults with hearing loss was associated with lesser healthcare utilization as compared to those who did not use the device [49]. HA use was also found to reduce risk of dementia, depression and falls related injury by up to 18% in the following three years after hearing loss diagnosis [50]. The current study only included short-term costs associated with hearing loss, thus potentially overestimating the ICER. It is therefore pertinent to study the longer-term effects of HA use and account for benefits in reducing risk of developing cognitive and mental conditions as well as preventable hospitalizations in Singapore.

With earlier screening of hearing loss in the adult population, future studies can explore ways to obtain a more representative study population of adults to investigate productivity gains in greater detail and over a longer period. Future research in Singapore can explore the potential use of dynamic patient flow models to simulate the various transition states for hearing loss and ongoing decisions problems in HA use to accurately evaluate the cost effectiveness of HA use for older adults with HI [28, 30, 51].

## Conclusions

This study suggests that fitting moderately impaired older adults with HA at a community-based MHC was cost-effective. Strategies such as promoting management of hearing loss at an earlier age, timely fitting and monitoring continued use of HA will be beneficial to both the individual as well as cost effectiveness of the device.

## Supplementary Information

**Additional file 1 Table S1**. Scoring Methodology for modified HUI-3. **Table S2**. Breakdown of Costs incurred at primary care or ENT specialist clinics. **Table S3**. Proportion of continued HA uptake after 1 year. **Fig. S1**. HUI-3 scores at all time-points.

**Additional file 2.** Direct and Indirect cost questionnaire.

## Data Availability

The datasets used and/or analysed during the current study are de-identified and available from the corresponding author on reasonable request.

## Abbreviations

HA: Hearing aids
MHC: Decibel hearing level (dB HL) mobile hearing clinic
QALY: Quality adjusted life years
ENT: Ear, nose and throat
SOC: Specialist outpatient clinic
HRQoL: Health related quality of life
HUI-3: Health utility index
CUA: Cost utility analysis
NICE: National institute for health and care excellence
PTA: Pure tone average
