# Supplementary material for: Cost-utility analysis of hearing aid device for older adults in the community: a delayed start study
Source: BMC Health Serv Res. 2020 Dec 1;20:1112. doi: 10.1186/s12913-020-05977-x (PMC7709244; doi:10.1186/s12913-020-05977-x)
Supplement: Supplementary file 1 — Additional file 1 Table S1. Scoring Methodology for modified HUI-3. Table S2. Breakdown of Costs incurred at primary care or ENT specialist clinics. Table S3. Proportion of continued HA uptake after 1 year. Fig. S1. HUI-3 scores at all time-points. [file 12913_2020_5977_MOESM1_ESM.docx]

**Additional file 1**

**Table S1: Scoring Methodology for modified HUI-3**

Based on the inclusion criteria of study, this population would not have used a HA at study entry. The table shows how the HUI-3 was modified to exclude HA related questions at baseline (pre-fitting timepoints). The modified HUI-3 was administered to the Not Fitted group at 3 months.

| **No.** | **Question** | **State 1** | **State 2** | **State 3** | **State 4** |
| --- | --- | --- | --- | --- | --- |
| 6 | During the past 4 weeks, have you been able to hear what is said in a group conversation with at least three other people without a hearing aid? | Y | N | N | N |
| 7 | Have you been able to hear what is said in a group conversation with at least three other people with a hearing aid? | - | - | - | - |
| 8 | During the past 4 weeks, have you been able to hear at all? | - | Y | N | Y |
| 9 | During the past 4 weeks, have you been able to hear what is said in a conversation, with one other person, in a quiet room without a hearing aid? | - | Y | - | N/DK |
| 10 | Have you been able to hear what is said in a conversation, with one other person, in a quiet room with a hearing aid? | - | - | - | - |
|  | Health State Profile | 1 | 4 | 6 | 5 |

Legend: Y: Yes; N: No; DK: Don’t Know

**Table S2: Breakdown of Costs incurred at primary care or ENT specialist clinics.**

| **Item** | **Costs in 2017 Singapore Dollar** |
| --- | --- |
| Norm costs for healthcare utilization   - ENT/private clinics - Polyclinic/primary care | - SGD 110 - SGD 18.60 |

**Table S3: Proportion of continued HA uptake after 1 year**

Response rate was 66%. 175 out of 264 Fitted Group responded.

| **Status** | **n (%)** |
| --- | --- |
| - Deceased - Lost - Not using HA - Continued using HA - Not Recoded | 3 (1.7%)  1 (0.6%)  30 (17.1%)  125 (71.4%)  16 (9.1%) |

**Figure S1: HUI-3 scores at all time points**

Fitted _3months_

Randomization at MHC

**HA & Rehab**

**No HA Fitting**

Fitted _Baseline_

Not Fitted _Baseline_

Not Fitted _3months_

**HA & Rehab**

Not Fitted _6months_

| **Group** | **HUI-3 scores Baseline (mean SD)** | **HUI-3 scores at 3 months (mean SD)** | **HUI-3 scores at 6 months (mean SD)** |
| --- | --- | --- | --- |
| **Fitted** | **0.6432 (0.222)** | **0.7657 (0.188)** | **-** |
| **Not fitted** | **0.6501 (0.250)** | **0.6487 (0.235)** | **0.7551 (0.193)** |

*Participants were randomised to Fitted or Not Fitted group at baseline (first visit to MHC). QALYs and total cost outcomes were compared between Fitted_3months_ and Not Fitted_3months_.*
