# Supplementary material for: Cost-utility analysis of hearing aid device for older adults in the community: a delayed start study
Source: BMC Health Serv Res. 2020 Dec 1;20:1112. doi: 10.1186/s12913-020-05977-x (PMC7709244; doi:10.1186/s12913-020-05977-x)
Supplement: Supplementary file 2 — Additional file 2. Direct and Indirect cost questionnaire. [file 12913_2020_5977_MOESM2_ESM.pdf]

Please tick (√) where applicable. **请在相应的空格上打(√)**

Questionnaire for patients concerning costs of hearing aid fitting

We ask you to answer the following questions. Please fill in the blanks or tick (√) where applicable

以下是有关助听器服务费用的问题，请回答所有问题，并在相应的空格上打(√)

To be filled by research assistants （由研究团队填妥）

Patient number: _______

Date of hearing aid evaluation: ____/____/______ (DD/MM/YYYY)

Follow-up at 3-month: ____/____/______ (DD/MM/YYYY)

Current date: ____/____/______ (DD/MM/YYYY)

Location of mobile hearing van: _____________________(Postal code:______________)

Postal code of residential address 住址邮区编号: ______________

1. How much time does it take for you to travel here (mobile van location)?

   您来听力流动诊所需要花多长的时间?

About 大約 _________ Hours 小時 _________ Minutes 分钟

2. How did you travel here? (you may choose more than one option)

   您搭乘什么交通工具来到这里(你可以选择多过一个)

	(√)	Cost of transport 交通费用
By bus 巴士	( )	$
By car 汽车	( )	-
By taxi 德士	( )	$
By MRT 地铁	( )	$
On foot or by bicycle 走路或是骑脚踏车	( )	-

3. What is your employment status over the last three months (since the last hearing aid fitting)?

   过去三个月，您从事什么工作(助听器配戴之后)

full-time or part-time 全职或兼职	( )

Please tick (√) where applicable. **请在相应的空格上打(√)**

NSF [1]<br>国民服役	( )	
Unemployed<br>暂无工作	( )	Proceed to question 7.<br>若选择此选项， 直接前往第七题
Retired<br>退休	( )	Proceed to question 7.<br>若选择此选项， 直接前往第七题
Student<br>学生	( )	Proceed to question 7.<br>若选择此选项,直接前往第七题

4. What is your current occupation? 您目前从事什么工作？_________________________

5. When visiting the mobile clinic over the past three months (inclusive of today), were you absent from work?
   过去三个月内(包括今天)当你前来过听力流动诊所复诊时，你有没有请假?

No 没有 ( )

Yes, about 有，大约 ________ total number of days 总共几天

________ hours per visit 小时(每次来访) ________ minutes per visit; or 分钟(每次来访)；或

( )	half-day off<br>半天	( )	one-day off<br>一天

6. If you are on sick leave in the past three months, what is the reason for the sick leave?
   过去三个月内您有没有请病假，因为什么理由请病假?

Hearing problem? 听力问题?	( )
Other disease, what? 其他疾病， 是什么？ ________________________	( )

Number of days on sick leave for hearing loss
总共几天(为了听力问题而请病假) ______________________

7. When visiting the mobile van over the past three months, do you usually have a caregiver who accompanies you here?
   过去三个月内(包括今天)当你前来过听力流动诊所复诊时，通常有没有人陪你一起来?

No.<br>没有	( )	Proceed to question 11.<br>若选择此选项，直接前往第十一题
Yes<br>有	( )	(family member/ relative/ domestic worker)<br>(家人/亲戚/帮佣)

[1] NSF – Full-time National Service 全职国民服役

Please tick (√) where applicable. **请在相应的空格上打(√)**

8. What is the employment status of your caregiver over the past three months?
过去三个月内, 照顾您的人从事什么工作?

full-time or part-time<br>全职或兼职	( )	
domestic helper<br>帮佣	( )	
NSF [2]<br>国民服役	( )	
Unemployed<br>暂无工作	( )	Proceed to question 12.<br>若选择此选项，直接前往第十二题
Retired<br>退休	( )	Proceed to question 12.<br>若选择此选项，直接前往第十二题
Student<br>学生	( )	Proceed to question 12.<br>若选择此选项，直接前往第十二题

9. Does your caregiver need to take time off from work to accompany you to the mobile van?

照顾您的人是否需要请假陪您来流动听力诊所?

No. 没有	( )						
Yes, about<br>有，大约	( )		total number of days<br>总共几天		hours per visit<br>小时(每次来访)		minutes per visit<br>分(每次来访)
or；或			total number of days<br>总共几天	( )	half-day off<br>半天	( )	one-day off<br>一天

10. What is your family member/ relative's current occupation (caregiver who came with you)? ____________________

过去三个月内,陪您前来诊所的家人/亲戚从事什么工作?

[2] NSF – Full-time National Service 全职国民服役

Please tick (√) where applicable. **请在相应的空格上打(√)**

11. How many times did you visit the following places over the last three months? (if zero times for all, end of survey).
过去三个月内, 您拜访过下列地方多少次？(如果没有到访过以下任何诊所，此问卷到此结束)

Ear Nose & Throat Specialist clinic<br>耳鼻喉专科诊所	________	times 次
Private clinic for hearing loss<br>私人诊所(针对听力问题)	________	times 次
Polyclinic visits for hearing loss<br>综合诊所(针对听力问题)	________	times 次

12. Which hospital/ clinic did you usually visit for hearing loss problem in the past three months?
过去三个月内, 因为听力问题， 您通常去哪一间医院/诊所?

Hospital<br>医院	________	Private clinic 私人诊所	________	Polyclinic<br>综合诊所	________

13. How much time does it usually take for you to travel to the clinic?
您去医院/诊所通常需要花多长的时间?

Ear Nose & Throat Specialist clinic<br>耳鼻喉专科诊所	________	hours per visit<br>小时(每次来访)	________	minutes per visit 分钟(每次来访)
Private clinic for hearing loss<br>私人诊所(针对听力问题)	________	hours per visit<br>小时(每次来访)	________	minutes per visit 分钟(每次来访)
Polyclinic visits for hearing loss<br>综合诊所(针对听力问题)	________	hours per visit<br>小时(每次来访)	________	minutes per visit 分钟(每次来访)

14. When visiting the clinic, are you usually absent from work?
当您到听力流动诊所复诊时，你通常有没有请假?

No. 没有	( )	
Yes 有	( )	fill in where applicable<br>如果有的话请填下列空格

Please tick (√) where applicable. **请在相应的空格上打**(√)

	No/ Yes 没有/ 有	-	(√)	-	(√)	-
Ear Nose & Throat Specialist clinic 耳鼻喉专科诊所		days[3] 天		hours per visit 小时(每次来访)		minutes per visit 分钟(每次来访)
	-		( )	half-day off 半天	( )	one-day off 一天
Private clinic for hearing loss 私人诊所(针对听力问题)		days[3] 天		hours per visit 小时(每次来访)		minutes per visit 分钟(每次来访)
	-		( )	half-day off 半天	( )	one-day off 一天
Polyclinic visits for hearing loss 综合诊所(针对听力问题)		days[3] 天		hours per visit 小时(每次来访)		minutes per visit 分钟(每次来访)
	-		( )	half-day off 半天	( )	one-day off 一天

15. How do you usually travel to the clinic?
    您通常搭乘什么交通工具去诊所?

	By bus 巴士 (√)	Cost of transport 交通费用	By car 汽车 (√)	By taxi 德士 (√)	Cost of transport 交通费用	By MRT 地铁 (√)	Cost of transport 交通费用	On foot or by bicycle 走路或是骑脚踏车 (√)
Ear Nose & Throat Specialist clinic 耳鼻喉专科诊所	( )	$	( )	( )	$	( )	$	( )
Private clinic for hearing loss 私人诊所(针对听力问题)	( )	$	( )	( )	$	( )	$	( )
Polyclinic visits for hearing loss 综合诊所(针对听力问题)	( )	$	( )	( )	$	( )	$	( )

[3] days – total number of days absent from work
总共请假几天

Please tick (√) where applicable. **请在相应的空格上打(√)**

16. If you were to visit a clinic for hearing loss problem, do you usually need a caregiver? Tick where applicable.
您因为听力问题而去诊所，通常有没有人陪你一起来?如果有的话请再想相应的空格打勾。

	No 没有 (√)	Yes, who? family member/ relative 有,(家人/亲戚) (√)	Domestic helper(帮佣 (√)
Ear Nose & Throat Specialist clinic 耳鼻喉专科诊所	( )	( )	( )
Private clinic for hearing loss 私人诊所(针对听力问题)	( )	( )	( )
Polyclinic visits for hearing loss 综合诊所(针对听力问题)	( )	( )	( )

17. If you are accompanied by caregiver, does he/she usually have to be absent from work?
如果有人陪伴的话,他/她通常有没有请假?

No. 没有	( )	
Yes 有	( )	fill in where applicable 如果有的话请填下列空格

	No/ Yes 有 /没有	-	(√)	-	(√)	-
Ear Nose & Throat Specialist clinic 耳鼻喉专科诊所		days[4] 天		hours per visit 小时(每次来访)		minutes per visit 分钟(每次来访)
	-		( )	half-day off 半天	( )	one-day off 一天
Private clinic for hearing loss 私人诊所(针对听力问题)		days[3] 天		hours per visit 小时(每次来访)		minutes per visit 分钟(每次来访)
	-		( )	half-day off 半天	( )	one-day off 一天
Polyclinic visits for hearing loss 综合诊所(针对听力问题)		days[3] 天		hours per visit 小时(每次来访)		minutes per visit 分钟(每次来访)
	-		( )	half-day off 半天	( )	one-day off 一天

Thank you! That ends this set of questionnaire.谢谢！问卷到此结束

[4] days – total number of days absent from work
总共请假几天
